# Metastatic heterogeneity in pancreatic cancer: mechanisms and opportunities for targeted intervention

**DOI:** 10.1172/JCI191943

**Published:** 2025-07-15

**Authors:** Ravikanth Maddipati

**Affiliations:** 1 Department of Internal Medicine, University of Texas Southwestern Medical Center, Dallas, Texas, USA.; 2 Veterans Administration North Texas Health Care System, Dallas, Texas, USA.; 3 Children’s Medical Center Research Institute and; 4 Harold C. Simmons Comprehensive Cancer Center, University of Texas Southwestern Medical Center, Dallas, Texas, USA.

## Abstract

Pancreatic ductal adenocarcinoma (PDAC) remains among the most lethal cancers, with metastasis as the primary driver of mortality. While metastatic mechanisms are shared across malignancies, PDAC metastasis poses unique therapeutic challenges due to the presence of extensive tumor heterogeneity, desmoplasia, and immunosuppression — features that enable diverse migratory behaviors and therapeutic resistance. Recent advances have shown that metastatic progression in PDAC emerges from dynamic interactions between tumor cell–intrinsic and microenvironmental factors, each adapting to evolving stressors throughout the metastatic cascade. In the primary tumor, genomic instability and epigenetic reprogramming generate subclones with heightened invasive potential, while dense stromal reactions and myeloid-dominated immune suppression facilitate escape. During circulation, PDAC cells employ distinctive survival strategies through homotypic clustering and heterotypic interactions with blood components. At distant sites, PDAC cells adapt to organ-specific microenvironments through context-dependent metabolic and immune modulation, resulting in phenotypes that diverge from the primary tumor. In this Review, we examine how tumor-stroma crosstalk mechanisms shape metastatic progression in PDAC, provide a framework for understanding why conventional therapies often fail against metastatic disease, and highlight emerging opportunities for stage- and site-specific therapeutic interventions that target these unique adaptations.

## Introduction

Metastasis remains the primary driver of mortality in most solid tumors (1). In pancreatic ductal adenocarcinoma (PDAC), metastasis presents with particularly aggressive features that distinguish it from other malignancies. While most cancer types show latency in metastatic development, PDAC exhibits an accelerated trajectory — over half of patients present with metastases at diagnosis, compared with approximately one-fifth in colorectal cancer (2). Even after complete surgical resection, 75%–80% of patients develop metastatic recurrence within five years, with spread to the liver (70%–80%), peritoneum (25%–30%), lung (15%–20%), and lymph nodes (15%–20%). This aggressive metastatic behavior largely explains why PDAC maintains one of the lowest five-year survival rates (12%) among all malignancies.

**Figure 1:**
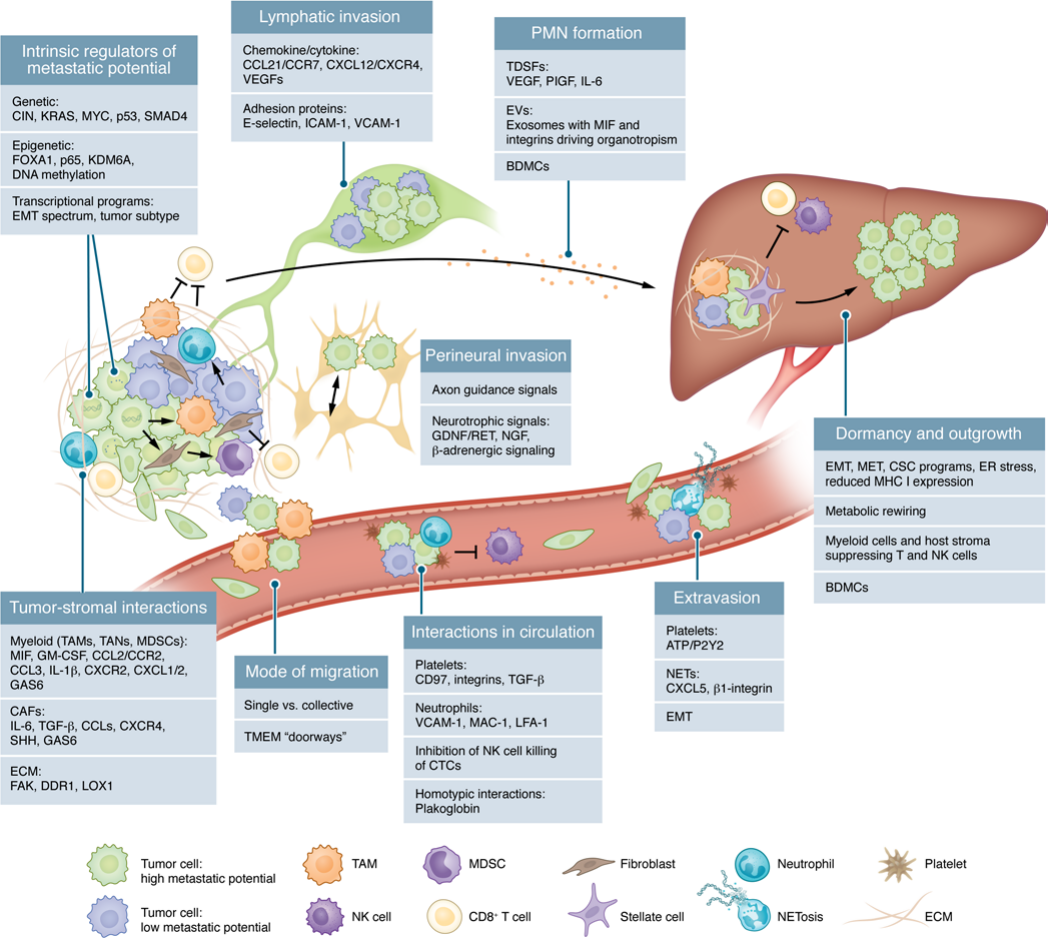
Tumor cell–intrinsic and –extrinsic regulation of the metastatic cascade. The schematic highlights key factors implicated in regulating metastasis across multiple cancers, including PDAC. Metastasis unfolds through a series of sequential steps — local invasion, intravasation, circulation, extravasation, and eventual outgrowth, each imposing distinct selective pressures on tumor cells. Intrinsic factors such as MYC or KRAS amplifications, epigenetic changes, and EMT states endow cells with invasive capabilities and enable remodeling of the TME. By secreting cytokines, chemokines, and extracellular vesicles, these invasive tumor cells recruit or reprogram myeloid cells, fibroblasts, and the ECM to facilitate local tissue invasion. Once in circulation, tumor cells travel individually or in clusters, influenced in part by whether they undergo “classical” versus partial EMT. Platelets and neutrophils also protect CTCs from immune attack (e.g., by NK cells), while cluster formation mitigates exposure to shear stress and metabolic changes (e.g., increased ROS). Dissemination can occur through lymphatic routes or along nerves, guided by specific stromal interactions. Upon reaching distant sites, tumor cells extravasate via leaky capillaries, aided by ongoing interactions with platelets and neutrophils. However, these new environments can be hostile. To overcome local barriers, tumor cells may precondition “premetastatic niches” through tumor-derived factors that induce immunosuppression and ECM remodeling. Dormancy can further enhance survival, allowing cells to adapt gradually. Many disseminated cells never progress, but others resume proliferation in response to external signals and by leveraging their intrinsic programs (e.g., EMT states, metabolic rewiring). Ultimately, site-specific interactions between tumor cells and the surrounding tissue microenvironment dictate outgrowth and metastatic heterogeneity. MET, mesenchymal-epithelial transition; PIGF, placental growth factor.

Metastasis arises from a complex series of steps — local invasion, intravasation, survival in circulation, and colonization of distant organs (Figure 1) (3). During each step, tumor cells encounter various selective pressures. How effectively they navigate these challenges depends on the dynamic interplay between tumor cell–intrinsic features (e.g., genomic, transcriptional, and epigenetic) and extrinsic microenvironmental influences (4). Viewed through the lens of Paget’s “seed and soil” hypothesis, evidence suggests that while properties of the seed (tumor cell) and soil (tissue environment) are crucial for establishing metastasis, each continuously adapts to influence one another (5). These tumor-stroma interactions give rise to variability in invasive behaviors of tumor cells and how they adapt to new tissue environments. This “metastatic heterogeneity” results in metastases with phenotypes that can vary within and between patients, often diverging from the primary tumor and rendering therapies less effective in these contexts (6).

In PDAC, this interplay between tumor-intrinsic and -extrinsic factors frequently reaches extremes, characterized by heightened cellular plasticity, profound immunosuppression, and a dense desmoplastic stroma (7). Such conditions within the primary tumor select for subpopulations with distinct metastatic competencies, capable of remodeling the local stroma, which in turn influences their invasive potential and mode of migration (8, 9). Similarly, at distant sites, PDAC cells can prime tissues to be more receptive for metastatic growth, yet their subsequent expansion is shaped by organ-specific microenvironments (10, 11). These interactions at each stage of metastasis drives the emergence of heterogeneous, stage- and site-specific metastatic phenotypes that may be strategically exploited for therapeutic gain.

In this Review, we highlight key advances in our understanding of how tumor cell–intrinsic and microenvironmental factors operate at each stage of the metastatic cascade. We examine how the interplay between these intrinsic and extrinsic elements gives rise to distinct metastatic phenotypes and identify potential therapeutic vulnerabilities that can be leveraged to treat metastasis.

## Origins of metastatic heterogeneity in the primary tumor

Metastasis initiates when tumor cells in the primary lesion gain the capacity to invade surrounding tissues to disseminate. This invasive behavior can vary substantially, within a tumor and across patients (6). For instance, in PDAC, some patients present with oligometastatic spread while others have substantial metastatic burden (12). Such variability arises from the underlying clonal diversity at the primary site, reflected in differences in the intrinsic invasive phenotypes of tumor cells and in how tumor cells orchestrate local microenvironmental conditions to enhance metastatic spread (13). In the following section, we examine how tumor-intrinsic and local tumor microenvironment (TME) factors in the primary tumor cooperate to govern the invasive potential of PDAC tumor cells (Figure 1).

## Tumor-intrinsic features regulating metastatic potential

Primary tumor development is viewed as an evolutionary process resulting from the accumulation of genetic and epigenetic changes that influence and are acted upon by the TME to drive growth (8). Applying this framework to metastasis, it was assumed that metastatic progression would likewise involve additional somatic mutations. However, early genome-wide sequencing efforts failed to pinpoint unique “metastasis genes” — genes that facilitate metastatic spread through mechanisms distinct from their function(s) in primary tumor growth (14). However, these initial studies were focused on identifying specific nucleotide mutations rather than broader structural changes to the genome. Subsequent studies have demonstrated that chromosomal instability (CIN) resulting in copy number alterations (CNAs) and structural rearrangements are pervasive in PDAC, where they drive malignant transformation and lay the groundwork for metastatic progression (12, 15–18).

Evidence suggests that both CIN and CNAs in specific oncogenes drive metastatic competency. For instance, CIN can directly promote tumor cell invasion by activating cytosolic DNA–sensing and –associated immune signaling pathways (15). In PDAC, CIN facilitates dissemination through STING-dependent mechanisms (19). Beyond these direct effects, CIN also provides a fertile genetic landscape for metastatic evolution, allowing for the development of subclones with varied invasive potential. Genomic analyses of matched primary and metastatic lesions revealed that metastases frequently develop from distinct subpopulations, with enriched CNAs affecting oncogenic drivers (12, 18). Specific mutations and corresponding CNAs appear to synergistically enhance metastatic potential. For example, amplifications in mutant KRAS alleles or the MYC oncogene display heightened downstream signaling, beyond what mutations alone confer (12, 17, 18). Loss-of-function alterations in tumor suppressors such as SMAD4 or TP53 further enhance invasiveness, likely by promoting genomic instability that cultivates the development of prometastatic subclones (20, 21). Thus, following the acquisition of the mutated oncogenes, continued tumor evolution and subsequent development of CNAs in these oncogenes facilitates metastasis. Taken together, these findings suggest that rather than acquiring new somatic mutations, tumor cells achieve metastatic competency by amplifying preexisting oncogenic programs through CNAs and altered gene dosage.

In conjunction with genetic changes, recent studies have shown that epigenetic mechanisms — including chromatin remodeling, histone modifications, and DNA methylation — also shape the metastatic potential of PDAC cells (22). Dysregulation in histone modifications can reconfigure chromatin landscapes, enabling activation of metastatic transcriptional programs (23). In PDAC, decreased repressive H3K9 methylation and increased H3K27 acetylation in chromatin regions enriched for gene networks that enhance invasion (23). Unlike irreversible genomic mutations, these histone modifications are reversible, allowing tumor cells to reprogram their transcriptomes in response to metastatic pressures. This can occur through enhancer reconfiguration via histone “readers” and “writers” (24). For instance, loss of the demethylase KDM6A accelerates PDAC progression, whereas low expression of the coactivator p300 correlates with heightened metastatic potential (25, 26). Similarly, epigenetic reprogramming carried out by transcription factors (TFs) can also remodel chromatin landscapes to regulate invasive behavior. The pioneer TF FOXA1 was found to promote anchorage-independent growth and invasion, while aberrant expression of p63 activated a squamous transcriptional program linked to metastasis (27, 28). Moreover, large-scale profiling studies have revealed that altered DNA methylation patterns can reinforce these transcriptional states, although direct causal links to metastasis remain to be delineated (29–31).

Genetic and epigenetic changes converge on tumor-intrinsic transcriptional programs that can influence metastatic behavior. Transcriptional profiling has established distinct molecular subtypes in PDAC, which can be broadly classified into classical and basal-like, with some tumors exhibiting hybrid features (16, 32–34). These subtypes display distinct molecular characteristics associated with metastatic potential. Major allelic imbalances in KRAS favoring the mutant allele occur more frequently in metastatic disease (29% versus 4% in primary tumors) and are enriched in basal subtypes (16). Basal-like tumors show activation of EMT, MYC, and cell cycle pathways linked to invasiveness (32). These molecular differences correlate with clinical outcomes, as patients with basal-like PDAC show worse survival compared with those with classical subtypes (5.7 versus 10.0 months) (33). Metastatic subtype distribution also varies by organ site, with classical subtypes enriched in lung and basal subtypes in liver metastases (35). This correlates with distinct gene expression programs, where liver-tropic tumors show heightened replication stress, while lung-tropic tumors display lower replication stress and enhanced T cell clonal responses (36). While these observations suggest an association between subtype and metastatic propensity, this relationship is complex, with both subtypes exhibiting features that enhance metastasis. Preclinical studies indicate that classical tumors may exhibit higher metastatic efficiency through formation of circulating tumor cell (CTC) clusters (discussed below) (37). Additionally, epithelial-like states characteristic of classical tumors may facilitate colonization. Furthermore, tumors can undergo subtype switching during progression, and primary lesions can contain mixed subtypes, complicating interpretations of subtype-metastasis relationships (35). Despite these complexities, determining the subtype of metastatic lesions remains clinically relevant, as basal-like and classical tumors demonstrate differential therapeutic sensitivities that could guide treatment selection (38, 39).

## Epithelial-mesenchymal transitions influence invasive phenotypes

A fundamental requirement for tumor invasion is phenotypic plasticity, enabling tumor cells to adapt to the diverse pressures of the metastatic cascade (1). This plasticity arises from a combination of intrinsic factors (genomic and epigenetic states) and extrinsic cues from the TME, which converge on transcriptional programs that enhance invasiveness. Epithelial-mesenchymal transition (EMT) is arguably the best-characterized mechanism regulating tumor invasion (40). Initially recognized as a developmental process, EMT is coopted by cancer cells to suppress epithelial traits and acquire mesenchymal characteristics, including motility and invasive behavior. However, the necessity of EMT for metastasis has been the subject of debate (41, 42). While multiple genetic models find EMT features throughout PDAC progression, some lineage-tracing studies suggest EMT might be dispensable for metastatic spread in certain contexts. However, recent dynamic lineage-tracing and ablation approaches demonstrate that EMT programs are indeed indispensable for tumor progression and also promote genomic instability (43). While its precise role in human metastasis remains unclear, due to lack of specific markers and difficulty in distinguishing fibroblasts from EMT cells in biopsies, EMT-associated transcriptional programs are consistently enriched in aggressive metastatic disease (9, 32, 35).

Traditionally, EMT is viewed as being driven at the transcriptional level by TFs (e.g., TWIST, SNAIL, ZEB1, PRRX1) and associated miRNAs that downregulate epithelial genes (such as those encoding E-cadherin, EPCAM, and claudins) while upregulating mesenchymal markers (N-cadherin, αSMA, vimentin) (44, 45). However, a growing body of work indicates that EMT spans a continuum of states, from fully mesenchymal phenotypes (“classical EMT”) to hybrid or “partial” EMT states in which cells retain elements of both epithelial and mesenchymal identity (40). In contrast to classical EMT, wherein epithelial proteins are regulated at the transcriptional level, partial EMT can be regulated by posttranscriptional mechanisms — such as cadherin internalization — and may endow cells with greater phenotypic flexibility, including the ability to revert via mesenchymal-epithelial transition (MET) at distant organ sites. EMT can be broadly viewed as a plastic program integrating genetic, epigenetic, and stromal signals to facilitate invasion (37, 46, 47).

A principal regulator of EMT in PDAC is extrinsic signals from the TME (45). As PDAC tumors expand, stromal components — including cancer-associated fibroblasts (CAFs), inflammatory cells, and an altered extracellular matrix (ECM) — release factors (e.g., TGF-β, WNT ligands, inflammatory cytokines) that stimulate EMT-TF networks. For example, GAS6 from the TME can activate AXL/TBK1 signaling in PDAC tumor cells to promote EMT and metastasis (48, 49). In addition, differences in the mechanical properties, ECM composition, and metabolic conditions across the tumor also influence the degree of EMT activation (45). For instance, elevated mechanical stress, high concentrations of matricellular proteins, or hypoxic signals can enhance EMT (50). Although most PDAC cells can undergo EMT when exposed to potent stimuli such as TGF-β, intrinsic differences exist in the extent and type of EMT they exhibit. In PDAC, some tumor cells adopt classical, whereas others follow a partial EMT program (37). This is partly determined by epigenetic modifications that regulate accessibility of EMT-related genes (51). Additionally, increased gene dosage of oncogenes (*KRAS*, *MYC*) or loss of tumor suppressors (*SMAD4*) can enhance activation of EMT and mesenchymal phenotypes (18, 32, 52, 53). Additionally, the specific mechanisms regulating EMT may also influence organotropism (i.e., organ-specific metastasis preferences). EMT associated with loss of p120 catenin has been linked to an increased propensity for lung metastasis, whereas stabilization of epithelial states facilitates liver colonization in PDAC (46, 54). Together, the local TME and intrinsic molecular landscape of tumor cells converge to regulate EMT states and invasiveness in PDAC.

The spectrum of EMT states present across tumors gives rise to diverse migratory and metastatic outcomes in PDAC (Figure 1). Tumor cells that undergo a full EMT often disseminate as single, highly motile cells, resulting in monoclonal metastatic lesions. By contrast, partial EMT states enable collective cell migration, in which tumor cells can travel as circulating clusters that seed metastases more efficiently and generate polyclonal metastases carrying more inherent tumor cell molecular diversity to distant sites (37, 55). While the precise contribution of each EMT state to metastatic burden remains unclear, human liquid biopsy analyses suggest both may play distinct but complementary roles. Single CTCs are typically more abundant, potentially reflecting the enhanced invasive capacity of cells undergoing complete EMT. Conversely, tumor clusters arising from partial EMT states are less frequent but correlate with worse clinical outcomes, suggesting they may constitute more potent metastatic seeds (56). The range of invasive phenotypes and plasticity associated with EMT drive molecular and phenotypic heterogeneity in metastases and as a result pose a substantial therapeutic challenge (57).

## Tumor-stroma interactions facilitate metastatic invasion

In conjunction with tumor-intrinsic factors, interactions of tumor cells with the TME are crucial to facilitating invasion (Figure 1) (4). The TME comprises a complex ecosystem of immune cells, fibroblasts, vasculature structures, and ECM (58). A principal mechanism by which the stroma enhances metastasis is through subversion of the host immune response by recruiting immunosuppressive myeloid cells and limiting activity of antitumor cytotoxic T lymphocytes (CTLs) (59). PDAC is often marked by an “immunologically cold” TME, with low effector T cell infiltration and abundant immunosuppressive populations, including tumor-associated macrophages (TAMs) (7). This immunosuppressive environment is established in part by tumor cell–intrinsic programs (Figure 1) that recruit suppressive myeloid populations, promote protumor inflammation, and dampen antigen presentation (12, 60, 61). Transcriptional and epigenetic heterogeneity in tumor cells further shape cytokine profiles and the TME (62). In murine PDAC, tumor cell–intrinsic regulation of CXCL1 was shown to be a determinant of a “non–T cell–inflamed” microenvironment that promoted metastasis (63). Additionally, the presence or absence of specific transcriptional subtypes can influence local immune infiltration (64). The net result is a TME where the immune system is largely coopted to support metastatic progression.

In the PDAC TME, myeloid cells are the most abundant and often function to suppress immune responses (65). TAMs, in particular, function as key facilitators of metastasis. TAMs span a continuum of phenotypes, from M1-like subtypes with antitumor activity to M2-like subtypes that promote immunosuppression (66). The PDAC TME is often marked by M2-like TAMs, which facilitate immune evasion by expressing T cell inhibitory ligands (e.g., PD-L1), secreting immunosuppressive mediators, and impeding CTL activity (65, 67, 68). Elimination of TAMs in PDAC mouse models was shown to reduce metastasis (12, 69). Similarly, MDSCs can impair T cell infiltration and promote immunosuppression in concert with M2-like TAMs (70). Tumor-associated neutrophils (TANs) have also been shown to be tumor promoting in PDAC and their ablation or inhibition of CXCR2 was found to abrogate metastasis (71). Together, these myeloid compartments reinforce a tolerogenic environment conducive to tumor invasion.

CAFs form the major nonimmune stromal population (7). CAFs produce and organize the ECM, creating a desmoplastic reaction that can physically limit T cell entry and activation (72, 73). CAFs also secrete cytokines and chemokines — such as IL-6 and TGF-β — that support invasion and immunosuppression (74). A subset of CAFs termed “antigen-presenting CAFs” (apCAFs) express MHC class II molecules to CD4^+^ T cells but lack costimulatory signals, resulting in activation of Tregs rather than effective antitumor responses and thus promoting metastatic progression (75). Paradoxically, CAFs can also exhibit tumor-restraining functions. Genetic or pharmacologic depletion of αSMA^+^ CAFs or Hedgehog signaling in PDAC, known to drive desmoplasia, results in more aggressive metastatic disease (76, 77). These findings highlight that CAFs and the signals that regulate them are not uniformly metastasis promoting; instead, distinct CAF subsets and molecular contexts determine whether they enhance or limit tumor spread.

Tumor-stroma interactions also remodel the ECM and vasculature to promote invasion (4, 78). TAMs, CAFs, and tumor cells secrete proteolytic enzymes — including metalloproteinases, cathepsins, and lysyl oxidases (LOX) — that reorganize ECM components (4, 79–83). PDAC expression of LOX can enhance collagen crosslinking and the ECM protein SPARC can alter collagen trafficking to weaken basement membrane barriers, creating channels for tumor cell migration (79, 80). Concomitantly, dense desmoplastic stroma and ongoing hypoxia drive proangiogenic factor (e.g., VEGF) secretion by tumor and stromal cells, resulting in a leaky vasculature more permissive to tumor cell intravasation (84). Additional stromal components can directly activate invasion. TGF-β from fibroblasts or EGF from macrophages can activate EMT and increase cell motility (85, 86). Tumor cells and macrophages can also interact directly to form TME of metastasis (TMEM) “doorways,” where macrophages facilitate transendothelial migration of tumor cells (Figure 1) (87). For example, MYC-amplified PDAC clones were found to increase TAM recruitment to create these TMEM sites (12). Collectively, these tumor-stroma interactions operate in tandem to promote invasion and dissemination in PDAC.

## Timing of dissemination

Another dimension of metastatic heterogeneity relates to the timing of dissemination — whether cells exit the primary site prior to full malignant transformation (early/parallel dissemination) or only after developing within an established tumor (late/serial dissemination) (88). The late dissemination model posits that metastasis occurs as a culmination of evolutionary selection within the primary tumor, where cells acquire successive mutations before gaining metastatic competency. This view is supported by genomic analyses of matched primary and metastatic lesions showing shared mutational landscapes, suggesting metastatic clones derive from advanced primary tumors (12, 89). Mathematical modeling based on these data estimates approximately 15 years from initiating mutations to metastatic dissemination, suggesting a substantial window for therapeutic intervention before metastasis. Conversely, the early dissemination model proposes that tumor cells can spread during premalignant stages. This is supported by lineage-tracing and clinical data showing cells can disseminate from preneoplastic pancreatic intraepithelial neoplasia lesions and pancreatic cysts, although their malignant potential remains uncertain (90, 91). Clinically, the frequent development of metastases (75%) within 5 years after R0 resection of localized PDAC, and rare cases of metastatic disease following pancreatectomy for benign conditions, suggest early dissemination may occur (92).

However, several methodological challenges complicate this debate. Genomic similarities between primary and metastatic lesions — often cited as evidence for late dissemination — could result from tumor self-seeding, where early disseminated cells return to the primary site (93). However, it remains unclear how early disseminated cells could acquire the same mutations present in late-stage tumors. While early disseminated cells might undergo parallel genomic evolution at distant sites, the chances of eventually converging on similar genomic alterations and transcriptional states would seem low (15, 16). Additionally, scarcity of early-stage PDAC specimens limits comprehensive genomic comparison across disease stages. From a clinical standpoint, timing of dissemination has important implications for treatment. If early dissemination predominates, then even seemingly localized disease may harbor occult metastatic cells, supporting aggressive adjuvant therapy after resection for R0 disease. This also supports implementation of lifelong monitoring of high-risk individuals with precursor lesions, incurring substantial costs and burden on patients. Conversely, if late dissemination is correct, then neoadjuvant approaches to reduce primary tumor burden before surgery may more effectively prevent metastasis. Thus, future studies clarifying the timing of dissemination may help tailor interventions, optimize follow-up intervals, and guide risk-based management of PDAC.

## CTCs engage diverse mechanism to disseminate

Once tumor cells breach the stromal barriers of the primary tumor, they enter the circulatory system as CTCs (94). Clinically, CTCs have emerged as prognostic markers in multiple cancers, including PDAC, where elevated pretreatment and posttreatment CTC counts are closely linked to worse survival and diminished therapy responses (95, 96).

Tumor cells most commonly disseminate through blood vessels and lymphatic channels but can also directly shed into the peritoneum or migrate along peripheral nerves (“perineural invasion”) (97). Although the precise reasons that some tumor cells favor a particular route remain unclear, evidence points to a confluence of chemokine/adhesion molecule profiles and local microenvironmental cues (Figure 1) (1, 98, 99). For instance, in PDAC, upregulated CCR7 and CXCR4 on tumor cells can promote lymph node invasion through binding to CCL21 and CXCL12 on lymphatic endothelium (99), whereas perineural invasion can involve axon guidance pathways (e.g., SEMA3D/PLXND1 and ROBO/SLIT2) and neurotrophic signals (e.g., GDNF/RET, NGF, and β-adrenergic signaling) shared between tumor cells and nerves (100–102).

The route by which tumor cells travel can influence CTC biology, including how tumor cells adapt to immune pressures and metabolic constraints during transit. For instance, lymph node metastases coincide with marked immunosuppression, including diminished CTL and NK cell function, reduced dendritic cell maturation, and expansion of Tregs (103). In melanoma, tumor cells exposed to the lymphatic microenvironment are protected from ferroptosis, which facilitates subsequent hematogenous spread (104). Additionally, the route of dissemination can also influence the mode of dissemination (55). Tumor cells can invade individually or collectively, giving rise to single-cell or clustered CTCs (105). We previously demonstrated that CTCs disseminating via ascitic fluid or lymphatic vessels were more frequently detected as multicellular clusters compared with those entering the bloodstream, suggesting that distinct routes of tumor cell spread may influence clonal composition at secondary sites (55).

During dissemination, CTCs must also navigate a complex circulatory environment where homo- and heterotypic interactions of CTCs impact their survival (Figure 1). Homotypic interactions in CTC clusters can protect against mechanical and oxidative stressors while also shielding cells from immune attack, enabling more efficient metastatic seeding (55, 106, 107). In contrast, single CTCs, while significantly greater in number, are more vulnerable to shear stress and immune-mediated killing — particularly by NK cells — which limits their metastatic efficiency. Among blood components, platelets have long been associated with tumor emboli formation (108). Platelets also protect CTCs from immune-mediated destruction, in part by inhibiting NK cell cytotoxicity (108–111). They also promote extravasation through release of ATP that binds endothelial P2Y2 receptors, loosening endothelial tight junctions and promote EMT through secretion of TGF-β (112, 113). Similarly, myeloid cells also influence CTC behavior, with neutrophils most frequently associated with CTCs (107, 114). By forming clusters with CTCs through adhesion molecules normally used for transendothelial migration, neutrophils enhance tumor cell extravasation and immune evasion (115). Moreover, under certain stimuli, neutrophils undergo a form of cell death termed NETosis, releasing neutrophil extracellular traps (NETs) composed of DNA and proteolytic enzymes (116). CTC-derived factors, such as CXCL5, IL-17, and extracellular vesicles, can trigger or amplify NET formation to trap tumor cells within the vasculature and promote extravasation (117–119). Taken together, the interplay between CTC-intrinsic and -extrinsic factors help tumor cells cope with the stresses of circulatory transit and contributes to the heterogeneity of metastatic dissemination.

## Acting at a distance: the premetastatic niche

Upon entry into circulation, the fate of tumor cells in distant organs depends partly on the receptiveness of the host tissue to support metastatic growth (120). Factors arising from the primary tumor can facilitate this process by priming the distant microenvironment, creating a premetastatic niche (PMN) (Figure 1) (120). Early murine studies in lung carcinoma and melanoma revealed that tumor-derived soluble factors (TDSFs) could mobilize bone marrow–derived cells (BMDCs) or induce secretion of inflammatory mediators by resident lung epithelia, to create favorable metastatic environments in host tissues (121, 122).

In PDAC, the PMN may facilitate and predict metastasis formation (123). The PMN arises through interdependent influences of TDSFs and reprogramming of stromal components at metastatic sites (Figure 1) (120). In PDAC, IL-6 production in the primary tumor stroma was shown to stimulate hepatocytes to release serum amyloid A (SAA), driving an immunosuppressive and fibrotic state conducive to metastatic outgrowth (124). Concomitantly, PMN assembly also depends on the recruitment of BDMCs to metastatic environments, as evidenced by infiltration of granulin-secreting monocytes into the liver triggering hepatic stellate cells (HSCs) to adopt a myofibroblastic phenotype, deposit ECM, and dampen local immune defenses (125). Notably, this priming often precedes overt metastasis development, possibly explaining why many patients present with advanced disease at diagnosis (124–126).

Extracellular vesicles (EVs) — particularly exosomes derived from tumor cells — act as potent mediators of PMN formation (120). EVs deliver an assortment of cargo (proteins, nucleic acids, and metabolites) that reshape stromal landscape in distal organs (127). In PDAC, exosomes bearing high levels of macrophage migration inhibitory factor (MIF) induce fibronectin deposition by HSCs, resulting in recruitment of BDMCs, and collectively generating a fibrotic and prometastatic environment (128). Exosomes can interfere with antitumor immunity by increasing suppressive myeloid populations, impairing NK cell function, and reprogramming stroma to bolster immune evasion (129, 130). Exosomes can also drive organotropism of CTCs (131). In breast and pancreatic cancer models, the integrin repertoires on exosomes help “pre-condition” and determine the site of metastatic colonization. For example, exosomes displaying integrin α6β4 or α6β1 predominantly facilitate lung metastasis, whereas exosomes bearing αvβ5 promote liver metastasis. Blocking these integrins diminishes exosome uptake and lowers metastatic burden in respective tissues (131).

Although the precise molecular networks governing PMN formation differ across tumor types, they broadly echo the dynamic interplay of tumor-intrinsic paracrine mediators and -extrinsic stromal factors that shape the invasion in the primary tumor by establishing a permissive “landing zone” at metastatic sites for tumor cells to seed and grow.

## Metastatic colonization

To colonize distant organs, tumor cells must extravasate from the vasculature, a process often facilitated by the mechanisms that supported their prior invasion and circulation, as reviewed earlier (Figure 1) (4). Although primary tumors shed cells continuously, only a small fraction successfully seed distant organs, and an even smaller subset progresses to form macroscopic metastatic lesions, underscoring the challenges disseminated tumor cells (DTCs) face when adapting to the unfamiliar conditions in metastatic sites (132).

After entering secondary tissues, DTCs face several possible fates. Most succumb to cell death or immune-mediated clearance. A subset may enter dormancy, persisting in a quiescent, nonproliferative state for variable intervals — a phase thought to confer a survival advantage by allowing cells to adapt to the metabolic and immune constraints of the new environment. Only the rare cells that eventually “awaken” and resume active proliferation progress to clinically overt metastases, driven by an interplay between cell-intrinsic and -extrinsic cues (133). In PDAC, dormancy is less clearly defined, partly because PDAC often presents at advanced stages and rarely exhibits the protracted latency intervals seen in other tumor types (92). Nonetheless, short-lived or context-dependent dormancy likely occurs in PDAC (134–136).

**Figure 2:**
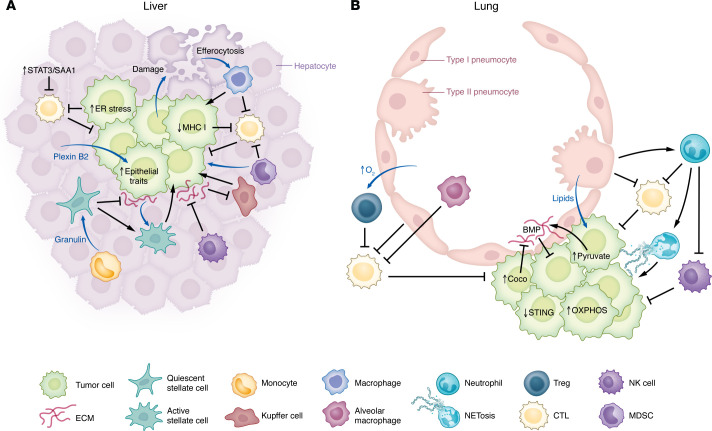
Site-specific tumor-stroma interactions regulating metastatic outgrowth. At distant sites, tumor cells encounter tissue microenvironments distinct from the primary tumor. Colonization depends partly on how they adapt to and reprogram local immune and metabolic niches. Here, we outline key tumor-intrinsic and -extrinsic interactions enabling liver and lung metastases, drawing on evidence from diverse cancer types, including PDAC. (**A**) Liver: Often considered tolerogenic, the liver epithelial and immune environments can be coopted by tumor cells to stimulate outgrowth. For instance, hepatocyte-derived plexin B2 activates epithelial programs in tumor cells, while induction of STAT3/SAA1 signaling in hepatocytes suppresses T cell responses. Tumor cell–induced damage of hepatocytes can trigger efferocytosis, which activates tumor-promoting myeloid cells. Antitumor Kupffer cells and NK cell responses are limited by tumor cells and local immunosuppression. Additional quiescent stellate cells that help maintain dormancy may be reprogrammed by monocyte-derived granulin or ECM stiffening into activated myofibroblasts that promote metastatic growth. (**B**) Lung: The lung harbors a distinct immune and metabolic niche compared with the liver. It contains type I and lipid-rich type II pneumocytes, alveolar macrophages, and immune defenses adapted to airborne pathogens and particulates. In PDAC, elevated immune infiltration in the lung may slow metastatic progression; however, multiple protumorigenic factors can facilitate growth, as described in other cancer types. Increased oxygen availability stimulates Tregs, while surfactant-derived lipids (e.g., palmitic acid) can fuel tumor growth. Both pneumocyte subtypes can suppress T cell activity and drive neutrophil recruitment, leading to NET formation or diminished NK cell function. Tumor cells further adapt by shifting to oxidative phosphorylation or downregulating STING to limit immune activation. Changes in pyruvate metabolism can influence collagen remodeling and Coco expression can counteract BMP signaling, both enabling metastatic expansion.

A central mechanism governing dormancy involves the phenotypic plasticity that enables DTCs to remain quiescent yet regain proliferative ability under the right stimuli (Figures 1 and 2) (1). Much like in primary lesions, EMT programs are a key mediator of this plasticity. Mesenchymal cells often cycle slowly and engage cancer stem cell (CSC) pathways to reduce metabolic demand and evade immune clearance (137–140). Transitioning back toward epithelial states (MET) is thought to promote outgrowth (141, 142). Cells in partial EMT states have greater flexibility in their EMT status and can revert to epithelial states more easily, supporting renewed proliferation (37). This flexibility often intersects with rewiring of DTC stress responses, metabolic, and immune programs to adapt to conditions at secondary sites (Figure 2) (143). Metabolically, dormancy is often associated with reduced glycolysis, increased oxidative phosphorylation (OXPHOS), and increased autophagy, which can promote mesenchymal phenotypes (23, 144). These metabolic shifts occur in part to minimize energy expenditure and limit reactive oxygen species (ROS) (143, 145). These adaptations can later be reversed to support growth in response to external cues. DTCs also modulate their immunogenicity through regulation of MHC-I expression (146, 147). For example, heightened ER-stress signaling in DTCs was associated with loss of MHC-I expression to escape T cell–mediated surveillance (147). Autocrine signals from DTCs may also alter metabolic programs or promote immune evasion by recruiting immunosuppressive myeloid cells or Tregs (148). In the context of breast cancer, these immune-mediated and metabolic shifts have been associated with STING activity, WNT pathways, and lactate production (149–151).

Local environmental cues can also be pivotal in regulating dormancy and outgrowth (Figure 2). For DTCs to form overt metastases, they must evade or suppress CTLs, NK cells, and other immune elements that constrain tumor expansion (148). Establishing the PMN is instrumental in this regard, as it primes local tissues for diminished immune clearance (10). Once tumor cells arrive, interactions with the resident stroma can perpetuate immunosuppression. For example, minor hepatic damage caused by PDAC cells can trigger efferocytosis by macrophages, reinforcing an immunosuppressive milieu (152). Additionally, metastases in regional lymph nodes may induce peripheral tolerance, and those in the liver can suppress CTL infiltration, collectively weakening antitumor immunity at more distant sites (153, 154).

In concert with local immune factors, metabolic cues such as hypoxia can induce dormancy by triggering cell cycle arrest, promoting EMT, and supporting autophagy to buffer against stress (155, 156). Shifts in nutrient availability likewise induce oxidative stress, prompting tumor cells to upregulate antioxidant pathways such as NRF2, which help promote metastasis (157). Interestingly, treating mice with the antioxidant *N*-acetylcysteine can increase metastasis in some models, implying that oxidative stress may restrict the expansion of dormant cells (145). However, in PDAC, ROS was found to enhance metastasis (158). These divergent responses to ROS likely reflect context-dependent effects influenced by the tumor’s genetic landscape, tissue of origin, and microenvironment. In PDAC, ROS can promote acquisition of mesenchymal phenotypes important for metastasis, while in melanoma and lung cancer, ROS appears to be detrimental to metastatic ability. Amid the hypoxia and limited nutrients, DTCs adjust their metabolism in concert with stromal cues. In PDAC liver metastases (Figure 2), HSCs can upregulate succinate dehydrogenase subunit B, biasing tumor cells toward an oxidative, quiescent phenotype (144). In contrast, inflammatory myofibroblasts stiffen the ECM and release inflammatory signals that drive proliferation and immune evasion (125). Beyond fibroblasts, the local epithelium also shapes DTC fate. In PDAC, hepatocyte-derived plexin 2 and IL-6/STAT3/SAA1 signaling facilitate liver colonization (124, 159).

The factors driving metastatic outgrowth also differ by organ site (Figure 2), a distinction that is clinically evident in PDAC, where patients with liver metastases have poorer survival than those with lung lesions (160, 161). We previously observed that clonal expansion patterns vary by metastatic location, indicating that tumor-intrinsic and -extrinsic signals differ by tissue context (55). A potential contributor to these differences is the immune and metabolic milieu in each organ. In the liver, NK cells and Kupffer cells can maintain tumor dormancy, but tumor-driven suppression of these defenses can trigger outgrowth (Figure 2) (162–164). In the lung, alveolar macrophages, Tregs, and neutrophils balance protection against airborne pathogens with immune tolerance, which can be coopted by DTCs to promote colonization (Figure 2) (165–167). However, an inflamed lung microenvironment in PDAC correlates with a more indolent course, whereas in other cancers it promotes aggressive spread, suggesting that tumor-intrinsic traits modulate site-specific immune effects (168). Similarly, distinct nutrient compositions also rewire DTC metabolism in a site-specific manner (169). For instance, breast cancer cells adapt to utilize the increased pyruvate and palmitic acid as nutrient source in the lung to proliferate (170, 171). Whether PDAC cells exploit these metabolic niches in a manner analogous to other malignancies and how these differences intersect with local immune regulation remains an open question.

## Conclusions and therapeutic implications

**Figure 3:**
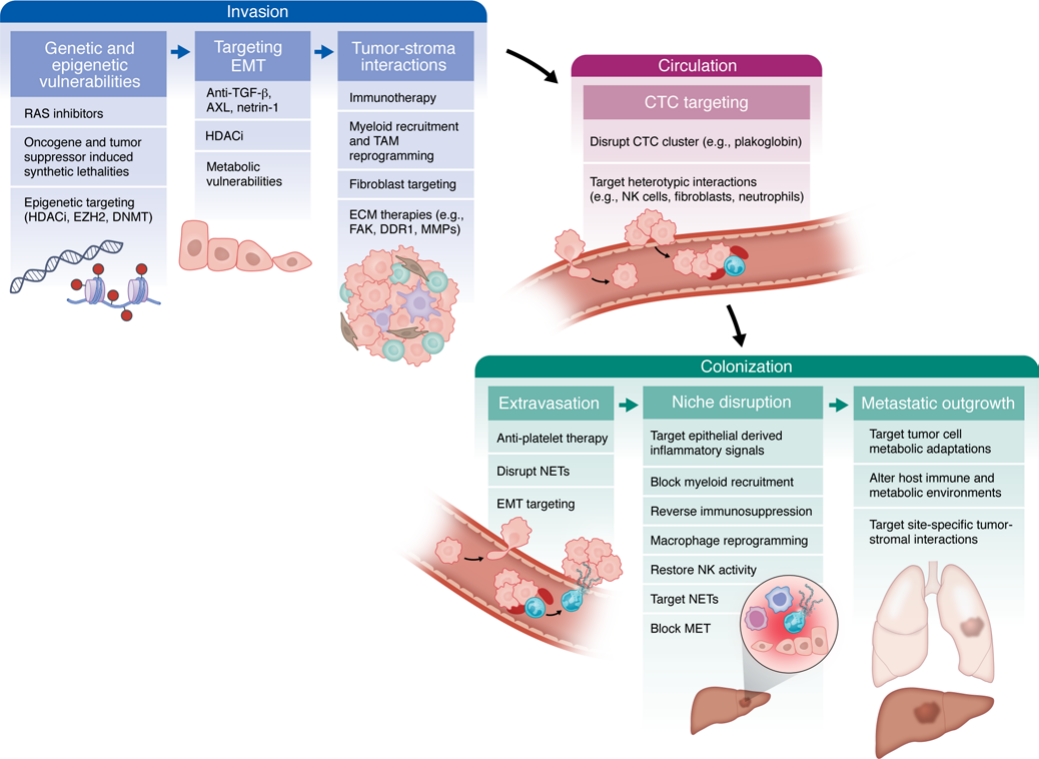
Stage-specific therapeutic vulnerabilities in metastasis. During metastasis, tumor cells adapt to immune, metabolic, and physical pressures at each step of the metastatic cascade (see Figure 1). Although these adaptations often yield more aggressive phenotypes, they also reveal vulnerabilities that can be therapeutically targeted. Here (and in Table 1), we propose a framework for designing stage- and site-specific therapies based on the dominant intrinsic (e.g., genetic, epigenetic) and extrinsic (e.g., TME-dependent) factors driving metastatic progression. Early invasion: Tumor cells acquiring MYC or KRAS amplifications gain heightened invasiveness, potentially amenable to novel RAS inhibitors or epigenetic therapies that exploit synthetic lethalities. Moreover, EMT induction often alters metabolic pathways and upregulates specific ligands/receptors (e.g., netrin-1, AXL), offering additional therapeutic points of intervention. Circulation: CTCs travel individually or in clusters that utilize platelet and neutrophil interactions for survival. Disrupting these heterotypic contacts, as well as limiting CTC cluster formation, may reduce metastatic dissemination. Metastatic colonization: Tumor-supportive niches and dormant-cell populations at distant sites constitute further therapeutic targets. Interventions aimed at inhibiting niche formation, reprogramming local stromal cells, or maintaining cancer cells in a dormant state could prevent full-blown metastatic outgrowth. HDACi, histone deacetylase inhibitor; EZH2, enhancer of zeste homolog 2; DNMT, DNA methyltransferase; MMPs, matrix metalloproteinases.

As revealed by large-scale multiomics efforts and preclinical models, PDAC metastasis is heterogeneous, influenced by tumor cell–intrinsic processes and the diverse microenvironments encountered at each stage of the metastatic cascade. Within the primary tumor, genomic and epigenetic dysregulation create populations with distinct invasive potentials, while reciprocal interactions with immune and nonimmune stromal elements further shape metastatic traits. Upon dissemination, tumor cells encounter and adapt to new cellular and metabolic environments that enable development of phenotypes distinct from the primary tumor (Figure 1). Rather than viewing this complexity as an insurmountable barrier, therapies should be tailored to disease stage and dominant mechanisms underlying metastasis in specific patient subgroups (Figure 3 and Table 1). For instance, classical tumor subtypes may benefit from approaches targeting collective cell migration and metabolic dependencies, while basal subtypes might respond better to strategies targeting EMT programs, genomic instability, and innate immune modulation. Similarly, disease stage provides another important framework for therapeutic stratification (Figure 3). For patients with R0 resection who remain at high risk for metastatic recurrence, therapies could focus on disrupting mechanisms that regulate dormancy or PMN formation. For those with established metastatic disease, approaches could be tailored to the extent of metastatic burden. Patients with oligometastatic disease may benefit from localized radiotherapy to prevent polyprogression, while those with widespread metastasis might require targeting organ-specific immune and metabolic milieus within which the metastases reside.

Moving forward, several advances are needed to realize the potential of stage- and site-specific antimetastatic therapies, especially considering current approaches to targeting tumor-stroma interactions in metastasis have had limited efficacy in PDAC (Table 2). First, improved biomarkers that predict metastatic recurrence in early-stage disease would enable more effective adjuvant therapy selection. Second, routine paired biopsies of primary tumors and metastases from multiple sites would enhance our understanding of how tumor cells adapt to different organ environments in each patient and enable therapies tailored to metastasis. Finally, clinical trials specifically designed to evaluate organ site–specific therapeutic approaches would determine whether targeting the unique adaptations of PDAC metastases in each organ could improve outcomes. Collectively, these insights underscore that effective treatment of metastasis will require multifaceted strategies that target site- and stage-specific factors ranging from tumor cell–intrinsic drivers of metastasis to the various microenvironmental niches encountered throughout the metastatic cascade.

## Figures and Tables

**Table 2:**
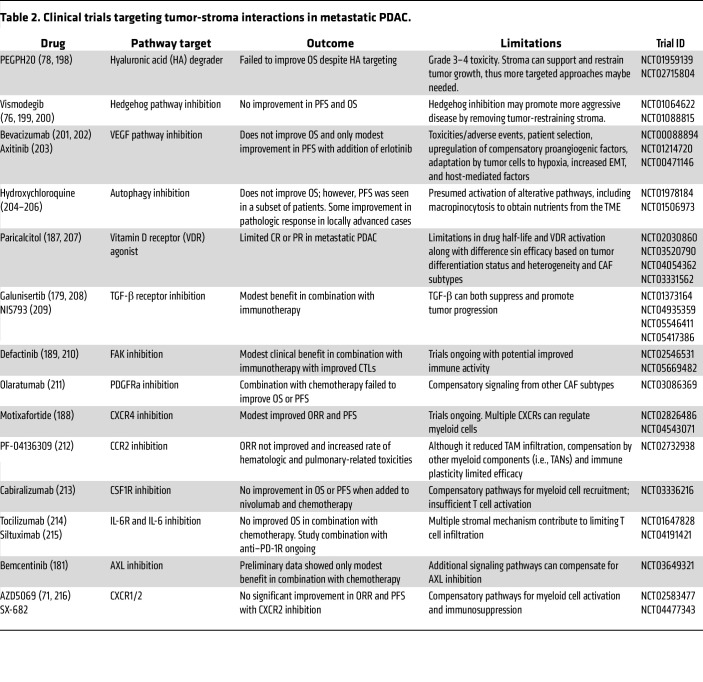
Clinical trials targeting tumor-stroma interactions in metastatic PDAC.

**Table 1:**
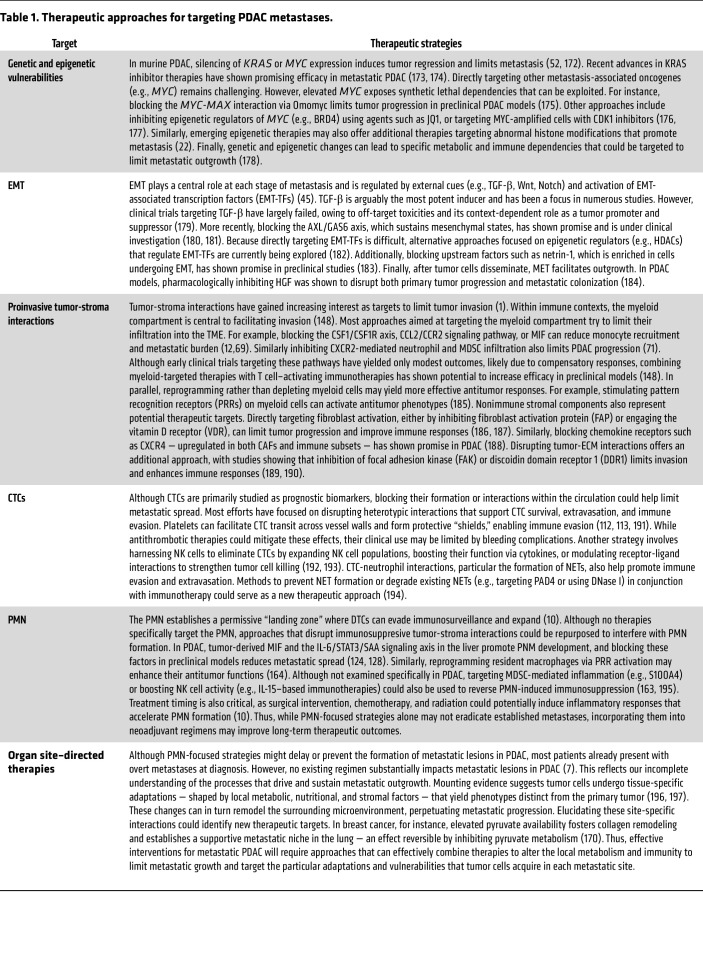
Therapeutic approaches for targeting PDAC metastases.

## Version 1 07/15/2025

Electronic publication

## References

[1] Lambert AW (2017). Emerging biological principles of metastasis. Cell.

[2] Rahib L (2014). Projecting cancer incidence and deaths to 2030: the unexpected burden of thyroid, liver, and pancreas cancers in the United States. Cancer Res.

[3] Poste G, Fidler IJ (1980). The pathogenesis of cancer metastasis. Nature.

[4] de Visser KE, Joyce JA (2023). The evolving tumor microenvironment: From cancer initiation to metastatic outgrowth. Cancer Cell.

[5] Fidler IJ (2003). The pathogenesis of cancer metastasis: the ‘seed and soil’ hypothesis revisited. Nat Rev Cancer.

[6] Vogelstein B (2013). Cancer genome landscapes. Science.

[7] Halbrook CJ (2023). Pancreatic cancer: advances and challenges. Cell.

[8] McGranahan N, Swanton C (2017). Clonal heterogeneity and tumor evolution: past, present, and the future. Cell.

[9] Peng J (2019). Single-cell RNA-seq highlights intra-tumoral heterogeneity and malignant progression in pancreatic ductal adenocarcinoma. Cell Res.

[10] Patras L (2023). Immune determinants of the pre-metastatic niche. Cancer Cell.

[11] Correia AL (2023). Locally sourced: site-specific immune barriers to metastasis. Nat Rev Immunol.

[12] Maddipati R (2022). *MYC* levels regulate metastatic heterogeneity in pancreatic adenocarcinoma. Cancer Discov.

[13] Karras P (2024). Decoding the interplay between genetic and non-genetic drivers of metastasis. Nature.

[14] Makohon-Moore AP (2017). Limited heterogeneity of known driver gene mutations among the metastases of individual patients with pancreatic cancer. Nat Genet.

[15] Bakhoum SF (2018). Chromosomal instability drives metastasis through a cytosolic DNA response. Nature.

[16] Notta F (2016). A renewed model of pancreatic cancer evolution based on genomic rearrangement patterns. Nature.

[17] Mueller S (2018). Evolutionary routes and KRAS dosage define pancreatic cancer phenotypes. Nature.

[18] Chan-Seng-Yue M (2020). Transcription phenotypes of pancreatic cancer are driven by genomic events during tumor evolution. Nat Genet.

[19] Wörmann SM (2021). APOBEC3A drives deaminase domain-independent chromosomal instability to promote pancreatic cancer metastasis. Nat Cancer.

[20] Muller PAJ (2009). Mutant p53 drives invasion by promoting integrin recycling. Cell.

[21] Iacobuzio-Donahue CA (2009). DPC4 gene status of the primary carcinoma correlates with patterns of failure in patients with pancreatic cancer. J Clin Oncol.

[22] Krauß L (2023). Epigenetic control of pancreatic cancer metastasis. Cancer Metastasis Rev.

[23] McDonald OG (2017). Epigenomic reprogramming during pancreatic cancer progression links anabolic glucose metabolism to distant metastasis. Nat Genet.

[24] Audia JE, Campbell RM (2016). Histone modifications and cancer. Cold Spring Harb Perspect Biol.

[25] Andricovich J (2018). Loss of KDM6A activates super-enhancers to induce gender-specific squamous-like pancreatic cancer and confers sensitivity to BET inhibitors. Cancer Cell.

[26] Mees ST (2010). EP300--a miRNA-regulated metastasis suppressor gene in ductal adenocarcinomas of the pancreas. Int J Cancer.

[27] Roe JS (2017). Enhancer reprogramming promotes pancreatic cancer metastasis. Cell.

[28] Somerville TDD (2018). TP63-mediated enhancer reprogramming drives the squamous subtype of pancreatic ductal adenocarcinoma. Cell Rep.

[29] Mishra NK, Guda C (2017). Genome-wide DNA methylation analysis reveals molecular subtypes of pancreatic cancer. Oncotarget.

[30] Thompson MJ (2015). Pancreatic cancer patient survival correlates with DNA methylation of pancreas development genes. PLoS One.

[31] Raman P (2018). Pancreatic cancer survival analysis defines a signature that predicts outcome. PLoS One.

[32] Bailey P (2016). Genomic analyses identify molecular subtypes of pancreatic cancer. Nature.

[33] Moffitt RA (2015). Virtual microdissection identifies distinct tumor- and stroma-specific subtypes of pancreatic ductal adenocarcinoma. Nat Genet.

[34] Raphael BJ (2017). Integrated genomic characterization of pancreatic ductal adenocarcinoma. Cancer Cell.

[35] Singh H (2024). Clinical and genomic features of classical and basal transcriptional subtypes in pancreatic cancer. Clin Cancer Res.

[36] Link JM (2025). Ongoing replication stress tolerance and clonal T cell responses distinguish liver and lung recurrence and outcomes in pancreatic cancer. Nat Cancer.

[37] Aiello NM (2018). EMT subtype influences epithelial plasticity and mode of cell migration. Dev Cell.

[38] Knox JJ (2024). Early results of the PASS-01 trial: Pancreatic adenocarcinoma signature stratification for treatment-01. J Clin Oncol.

[39] O’Kane GM (2020). GATA6 expression distinguishes classical and basal-like subtypes in advanced pancreatic cancer. Clin Cancer Res.

[40] Li W, Kang Y (2016). Probing the fifty shades of EMT in metastasis. Trends Cancer.

[41] Fischer KR et al (2015). Epithelial-to-mesenchymal transition is not required for lung metastasis but contributes to chemoresistance. Nature.

[42] Zheng X (2015). Epithelial-to-mesenchymal transition is dispensable for metastasis but induces chemoresistance in pancreatic cancer. Nature.

[44] Nieto MA (2016). EMT: 2016. Cell.

[45] Dongre A, Weinberg RA (2019). New insights into the mechanisms of epithelial-mesenchymal transition and implications for cancer. Nat Rev Mol Cell Biol.

[46] Reichert M (2018). Regulation of epithelial plasticity determines metastatic organotropism in pancreatic cancer. Dev Cell.

[47] Lüönd F (2021). Distinct contributions of partial and full EMT to breast cancer malignancy. Dev Cell.

[48] Arner EN (2024). AXL-TBK1 driven AKT3 activation promotes metastasis. Sci Signal.

[49] Cruz VH (2019). Axl-mediated activation of TBK1 drives epithelial plasticity in pancreatic cancer. JCI Insight.

[50] Ye X, Weinberg RA (2015). Epithelial-mesenchymal plasticity: a central regulator of cancer progression. Trends Cell Biol.

[51] Yuan S (2020). Global regulation of the histone mark H3K36me2 underlies epithelial plasticity and metastatic progression. Cancer Discov.

[52] Sodir NM (2020). MYC instructs and maintains pancreatic adenocarcinoma phenotype. Cancer Discov.

[53] Bardeesy N (2006). Smad4 is dispensable for normal pancreas development yet critical in progression and tumor biology of pancreas cancer. Genes Dev.

[54] Carstens JL (2021). Stabilized epithelial phenotype of cancer cells in primary tumors leads to increased colonization of liver metastasis in pancreatic cancer. Cell Rep.

[55] Maddipati R, Stanger BZ (2015). Pancreatic cancer metastases harbor evidence of polyclonality. Cancer Discov.

[56] Yeo D (2022). Exploring the clinical utility of pancreatic cancer circulating tumor cells. Int J Mol Sci.

[57] Papargyriou A (2025). Heterogeneity-driven phenotypic plasticity and treatment response in branched-organoid models of pancreatic ductal adenocarcinoma [published online December 10, 2024]. Nat Biomed Eng.

[58] Feig C (2012). The pancreas cancer microenvironment. Clin Cancer Res.

[59] Zhang Z (2023). Single-cell mapping reveals several immune subsets associated with liver metastasis of pancreatic ductal adenocarcinoma. Med.

[60] Pylayeva-Gupta Y (2012). Oncogenic Kras-induced GM-CSF production promotes the development of pancreatic neoplasia. Cancer Cell.

[61] Shchors K (2006). The Myc-dependent angiogenic switch in tumors is mediated by interleukin 1beta. Genes Dev.

[62] Balli D (2017). Immune cytolytic activity stratifies molecular subsets of human pancreatic cancer. Clin Cancer Res.

[63] Li J (2018). Tumor cell-intrinsic factors underlie heterogeneity of immune cell infiltration and response to immunotherapy. Immunity.

[64] Raghavan S (2021). Microenvironment drives cell state, plasticity, and drug response in pancreatic cancer. Cell.

[65] DeNardo DG, Ruffell B (2019). Macrophages as regulators of tumour immunity and immunotherapy. Nat Rev Immunol.

[66] Jablonski KA (2015). Novel markers to delineate murine M1 and M2 Macrophages. PLoS One.

[67] Bayne LJ (2012). Tumor-derived granulocyte-macrophage colony-stimulating factor regulates myeloid inflammation and T cell immunity in pancreatic cancer. Cancer Cell.

[68] Candido JB (2018). CSF1R^+^ macrophages sustain pancreatic tumor growth through T cell suppression and maintenance of key gene programs that define the squamous subtype. Cell Rep.

[69] Mitchem JB (2013). Targeting tumor-infiltrating macrophages decreases tumor-initiating cells, relieves immunosuppression, and improves chemotherapeutic responses. Cancer Res.

[70] Stromnes IM (2014). Targeted depletion of an MDSC subset unmasks pancreatic ductal adenocarcinoma to adaptive immunity. Gut.

[71] Steele CW (2016). CXCR2 inhibition profoundly suppresses metastases and augments immunotherapy in pancreatic ductal adenocarcinoma. Cancer Cell.

[72] Harper J, Sainson RCA (2014). Regulation of the anti-tumour immune response by cancer-associated fibroblasts. Semin Cancer Biol.

[73] Whittle MC, Hingorani SR (2019). Fibroblasts in pancreatic ductal adenocarcinoma: biological mechanisms and therapeutic targets. Gastroenterology.

[74] Öhlund D (2017). Distinct populations of inflammatory fibroblasts and myofibroblasts in pancreatic cancer. J Exp Med.

[75] Huang H (2022). Mesothelial cell-derived antigen-presenting cancer-associated fibroblasts induce expansion of regulatory T cells in pancreatic cancer. Cancer Cell.

[76] Rhim AD (2014). Stromal elements act to restrain, rather than support, pancreatic ductal adenocarcinoma. Cancer Cell.

[77] Özdemir BC (2014). Depletion of carcinoma-associated fibroblasts and fibrosis induces immunosuppression and accelerates pancreas cancer with reduced survival. Cancer Cell.

[78] Provenzano PP (2012). Enzymatic targeting of the stroma ablates physical barriers to treatment of pancreatic ductal adenocarcinoma. Cancer Cell.

[79] Morrissey MA (2016). SPARC promotes cell invasion in vivo by decreasing type IV collagen levels in the basement membrane. PLoS Genet.

[80] Miller BW (2015). Targeting the LOX/hypoxia axis reverses many of the features that make pancreatic cancer deadly: inhibition of LOX abrogates metastasis and enhances drug efficacy. EMBO Mol Med.

[81] Olson OC, Joyce JA (2015). Cysteine cathepsin proteases: regulators of cancer progression and therapeutic response. Nat Rev Cancer.

[82] Akkari L (2014). Distinct functions of macrophage-derived and cancer cell-derived cathepsin Z combine to promote tumor malignancy via interactions with the extracellular matrix. Genes Dev.

[83] Maller O (2021). Tumour-associated macrophages drive stromal cell-dependent collagen crosslinking and stiffening to promote breast cancer aggression. Nat Mater.

[84] De Palma M (2017). Microenvironmental regulation of tumour angiogenesis. Nat Rev Cancer.

[85] Zavadil J, Böttinger EP (2005). TGF-beta and epithelial-to-mesenchymal transitions. Oncogene.

[86] Lewis CE, Pollard JW (2006). Distinct role of macrophages in different tumor microenvironments. Cancer Res.

[87] Ginter PS (2019). Tumor microenvironment of metastasis (TMEM) doorways are restricted to the blood vessel endothelium in both primary breast cancers and their lymph node metastases. Cancers (Basel).

[88] Klein CA (2009). Parallel progression of primary tumours and metastases. Nat Rev Cancer.

[89] Yachida S (2010). Distant metastasis occurs late during the genetic evolution of pancreatic cancer. Nature.

[90] Rhim AD (2012). EMT and dissemination precede pancreatic tumor formation. Cell.

[91] Franses JW (2018). Improved detection of circulating epithelial cells in patients with intraductal papillary mucinous neoplasms. Oncologist.

[92] Groot VP (2018). Patterns, timing, and predictors of recurrence following pancreatectomy for pancreatic ductal adenocarcinoma. Ann Surg.

[93] Kim MY (2009). Tumor self-seeding by circulating cancer cells. Cell.

[94] Kang Y, Pantel K (2013). Tumor cell dissemination: emerging biological insights from animal models and cancer patients. Cancer Cell.

[95] Pang TCY (2021). Circulating tumour cells in pancreatic cancer: A systematic review and meta-analysis of clinicopathological implications. Pancreatology.

[96] Ring A et al (2023). Biology, vulnerabilities and clinical applications of circulating tumour cells. Nat Rev Cancer.

[97] DiMagno EP (1999). AGA technical review on the epidemiology, diagnosis, and treatment of pancreatic ductal adenocarcinoma. American Gastroenterological Association. Gastroenterology.

[98] Paduch R (2016). The role of lymphangiogenesis and angiogenesis in tumor metastasis. Cell Oncol (Dordr).

[99] Fink DM (2016). The lymphatic system and pancreatic cancer. Cancer Lett.

[100] Jurcak NR (2019). Axon guidance molecules promote perineural invasion and metastasis of orthotopic pancreatic tumors in mice. Gastroenterology.

[101] Li J (2021). Cellular and molecular mechanisms of perineural invasion of pancreatic ductal adenocarcinoma. Cancer Commun (Lond).

[102] Göhrig A (2014). Axon guidance factor SLIT2 inhibits neural invasion and metastasis in pancreatic cancer. Cancer Res.

[103] Delclaux I (2024). The tumor-draining lymph node as a reservoir for systemic immune surveillance. Trends Cancer.

[104] Ubellacker JM (2020). Lymph protects metastasizing melanoma cells from ferroptosis. Nature.

[105] Alix-Panabières C, Pantel K (2021). Liquid biopsy: from discovery to clinical application. Cancer Discov.

[106] Aceto N (2014). Circulating tumor cell clusters are oligoclonal precursors of breast cancer metastasis. Cell.

[107] Pereira-Veiga T (2022). Circulating tumor cell-blood cell crosstalk: Biology and clinical relevance. Cell Rep.

[109] Anvari S (2021). Interactions of platelets with circulating tumor cells contribute to cancer metastasis. Sci Rep.

[110] Erpenbeck L, Schön MP (2010). Deadly allies: the fatal interplay between platelets and metastasizing cancer cells. Blood.

[111] Liu X (2023). Immune checkpoint HLA-E:CD94-NKG2A mediates evasion of circulating tumor cells from NK cell surveillance. Cancer Cell.

[112] Schumacher D (2013). Platelet-derived nucleotides promote tumor-cell transendothelial migration and metastasis via P2Y2 receptor. Cancer Cell.

[113] Labelle M (2011). Direct signaling between platelets and cancer cells induces an epithelial-mesenchymal-like transition and promotes metastasis. Cancer Cell.

[114] Zhang Q (2021). Neutrophil-to-lymphocyte ratio correlates with prognosis and response to chemotherapy in patients with non-M3 *de novo* acute myeloid leukemia. Transl Cancer Res.

[115] Spicer JD (2012). Neutrophils promote liver metastasis via Mac-1-mediated interactions with circulating tumor cells. Cancer Res.

[116] Brinkmann V (2004). Neutrophil extracellular traps kill bacteria. Science.

[117] Deng J (2021). DDR1-induced neutrophil extracellular traps drive pancreatic cancer metastasis. JCI Insight.

[118] Zhang Y (2020). Interleukin-17-induced neutrophil extracellular traps mediate resistance to checkpoint blockade in pancreatic cancer. J Exp Med.

[119] Charles Jacob HK (2021). Modulation of early neutrophil granulation: the circulating tumor cell-extravesicular connection in pancreatic ductal adenocarcinoma. Cancers (Basel).

[120] Liu Y, Cao X (2016). Characteristics and significance of the pre-metastatic niche. Cancer Cell.

[121] Kaplan RN (2005). VEGFR1-positive haematopoietic bone marrow progenitors initiate the pre-metastatic niche. Nature.

[122] Hiratsuka S (2008). The S100A8-serum amyloid A3-TLR4 paracrine cascade establishes a pre-metastatic phase. Nat Cell Biol.

[123] Bojmar L (2024). Multi-parametric atlas of the pre-metastatic liver for prediction of metastatic outcome in early-stage pancreatic cancer. Nat Med.

[124] Lee JW (2019). Hepatocytes direct the formation of a pro-metastatic niche in the liver. Nature.

[125] Nielsen SR (2016). Macrophage-secreted granulin supports pancreatic cancer metastasis by inducing liver fibrosis. Nat Cell Biol.

[126] Seubert B (2015). Tissue inhibitor of metalloproteinases (TIMP)-1 creates a premetastatic niche in the liver through SDF-1/CXCR4-dependent neutrophil recruitment in mice. Hepatology.

[127] Hoshino A (2020). Extracellular vesicle and particle biomarkers define multiple human cancers. Cell.

[128] Costa-Silva B (2015). Pancreatic cancer exosomes initiate pre-metastatic niche formation in the liver. Nat Cell Biol.

[129] Zhao J (2019). Tumor-derived extracellular vesicles inhibit natural killer cell function in pancreatic cancer. Cancers (Basel).

[130] Jung T (2009). CD44v6 dependence of premetastatic niche preparation by exosomes. Neoplasia.

[131] Hoshino A (2015). Tumour exosome integrins determine organotropic metastasis. Nature.

[132] Luzzi KJ (1998). Multistep nature of metastatic inefficiency: dormancy of solitary cells after successful extravasation and limited survival of early micrometastases. Am J Pathol.

[133] Blasco MT (2022). Ecology and evolution of dormant metastasis. Trends Cancer.

[134] Hermann PC (2007). Distinct populations of cancer stem cells determine tumor growth and metastatic activity in human pancreatic cancer. Cell Stem Cell.

[135] Lin WC (2013). Dormant cancer cells contribute to residual disease in a model of reversible pancreatic cancer. Cancer Res.

[137] Lawson DA (2015). Single-cell analysis reveals a stem-cell program in human metastatic breast cancer cells. Nature.

[138] Dongre A (2021). Direct and indirect regulators of epithelial-mesenchymal transition-mediated immunosuppression in breast carcinomas. Cancer Discov.

[139] Tauriello DVF (2018). TGFβ drives immune evasion in genetically reconstituted colon cancer metastasis. Nature.

[140] Krebs AM (2017). The EMT-activator Zeb1 is a key factor for cell plasticity and promotes metastasis in pancreatic cancer. Nat Cell Biol.

[141] Bakir B (2020). EMT, MET, plasticity, and tumor metastasis. Trends Cell Biol.

[142] Padmanaban V (2019). E-cadherin is required for metastasis in multiple models of breast cancer. Nature.

[143] Ganguly K, Kimmelman AC (2023). Reprogramming of tissue metabolism during cancer metastasis. Trends Cancer.

[144] Fabian A (2019). Metastasis of pancreatic cancer: An uninflamed liver micromilieu controls cell growth and cancer stem cell properties by oxidative phosphorylation in pancreatic ductal epithelial cells. Cancer Lett.

[145] Piskounova E (2015). Oxidative stress inhibits distant metastasis by human melanoma cells. Nature.

[146] Agudo J (2018). Quiescent tissue stem cells evade immune surveillance. Immunity.

[147] Pommier A (2018). Unresolved endoplasmic reticulum stress engenders immune-resistant, latent pancreatic cancer metastases. Science.

[148] Blomberg OS (2018). Immune regulation of metastasis: mechanistic insights and therapeutic opportunities. Dis Model Mech.

[149] Hu J (2023). STING inhibits the reactivation of dormant metastasis in lung adenocarcinoma. Nature.

[150] Malladi S (2016). Metastatic latency and immune evasion through autocrine inhibition of WNT. Cell.

[151] Parida PK (2022). Metabolic diversity within breast cancer brain-tropic cells determines metastatic fitness. Cell Metab.

[152] Astuti Y (2024). Efferocytosis reprograms the tumor microenvironment to promote pancreatic cancer liver metastasis. Nat Cancer.

[153] Reticker-Flynn NE (2022). Lymph node colonization induces tumor-immune tolerance to promote distant metastasis. Cell.

[154] Yu J (2021). Liver metastasis restrains immunotherapy efficacy via macrophage-mediated T cell elimination. Nat Med.

[155] Fluegen G (2017). Phenotypic heterogeneity of disseminated tumour cells is preset by primary tumour hypoxic microenvironments. Nat Cell Biol.

[156] Martinez-Outschoorn UE (2010). Autophagy in cancer associated fibroblasts promotes tumor cell survival: Role of hypoxia, HIF1 induction and NFκB activation in the tumor stromal microenvironment. Cell Cycle.

[157] Wiel C (2019). BACH1 stabilization by antioxidants stimulates lung cancer metastasis. Cell.

[158] Cheung EC (2020). Dynamic ROS control by TIGAR regulates the initiation and progression of pancreatic cancer. Cancer Cell.

[159] Borrelli C (2024). In vivo interaction screening reveals liver-derived constraints to metastasis. Nature.

[160] Oweira H (2017). Prognostic value of site-specific metastases in pancreatic adenocarcinoma: A Surveillance Epidemiology and End Results database analysis. World J Gastroenterol.

[161] García-Mulero S (2020). Lung metastases share common immune features regardless of primary tumor origin. J Immunother Cancer.

[162] Zhang S (2023). Single cell transcriptomic analyses implicate an immunosuppressive tumor microenvironment in pancreatic cancer liver metastasis. Nat Commun.

[163] Correia AL (2021). Hepatic stellate cells suppress NK cell-sustained breast cancer dormancy. Nature.

[164] Thomas SK (2023). Kupffer cells prevent pancreatic ductal adenocarcinoma metastasis to the liver in mice. Nat Commun.

[165] Sharma SK (2015). Pulmonary alveolar macrophages contribute to the premetastatic niche by suppressing antitumor T cell responses in the lungs. J Immunol.

[166] Clever D (2016). Oxygen sensing by T cells establishes an immunologically tolerant metastatic niche. Cell.

[167] Albrengues J (2018). Neutrophil extracellular traps produced during inflammation awaken dormant cancer cells in mice. Science.

[168] Ho WJ (2021). Multi-omic profiling of lung and liver tumor microenvironments of metastatic pancreatic cancer reveals site-specific immune regulatory pathways. Genome Biol.

[169] Elia I (2018). Metabolic hallmarks of metastasis formation. Trends Cell Biol.

[170] Elia I (2019). Breast cancer cells rely on environmental pyruvate to shape the metastatic niche. Nature.

[171] Altea-Manzano P (2023). A palmitate-rich metastatic niche enables metastasis growth via p65 acetylation resulting in pro-metastatic NF-κB signaling. Nat Cancer.

[172] Collins MA (2012). Oncogenic Kras is required for both the initiation and maintenance of pancreatic cancer in mice. J Clin Invest.

[173] Kemp SB (2023). Efficacy of a small-molecule inhibitor of KrasG12D in immunocompetent models of pancreatic cancer. Cancer Discov.

[174] Wasko UN (2024). Tumour-selective activity of RAS-GTP inhibition in pancreatic cancer. Nature.

[175] Garralda E (2024). MYC targeting by OMO-103 in solid tumors: a phase 1 trial. Nat Med.

[176] Mazur PK (2015). Combined inhibition of BET family proteins and histone deacetylases as a potential epigenetics-based therapy for pancreatic ductal adenocarcinoma. Nat Med.

[177] Goga A (2007). Inhibition of CDK1 as a potential therapy for tumors over-expressing MYC. Nat Med.

[178] Wellenstein MD, de Visser KE (2018). Cancer-cell-intrinsic mechanisms shaping the tumor immune landscape. Immunity.

[179] Principe DR (2014). TGF-β: duality of function between tumor prevention and carcinogenesis. J Natl Cancer Inst.

[180] Zhang Y (2022). AXL inhibitor TP-0903 reduces metastasis and therapy resistance in pancreatic cancer. Mol Cancer Ther.

[181] Bhalla S, Gerber DE (2023). AXL inhibitors: status of clinical development. Curr Oncol Rep.

[182] Wawruszak A (2019). Histone deacetylase inhibitors and phenotypical transformation of cancer cells. Cancers (Basel).

[183] Lengrand J (2023). Pharmacological targeting of netrin-1 inhibits EMT in cancer. Nature.

[184] Takano S (2016). Prrx1 isoform switching regulates pancreatic cancer invasion and metastatic colonization. Genes Dev.

[185] Sun L (2021). Activating a collaborative innate-adaptive immune response to control metastasis. Cancer Cell.

[186] Melero I (2023). A first-in-human study of the fibroblast activation protein-targeted, 4-1BB agonist RO7122290 in patients with advanced solid tumors. Sci Transl Med.

[187] Sherman MH (2014). Vitamin D receptor-mediated stromal reprogramming suppresses pancreatitis and enhances pancreatic cancer therapy. Cell.

[188] Bockorny B (2020). BL-8040, a CXCR4 antagonist, in combination with pembrolizumab and chemotherapy for pancreatic cancer: the COMBAT trial. Nat Med.

[189] Wang-Gillam A (2022). Defactinib, pembrolizumab, and gemcitabine in patients with advanced treatment refractory pancreatic cancer: a phase I dose escalation and expansion study. Clin Cancer Res.

[190] Su H (2022). Collagenolysis-dependent DDR1 signalling dictates pancreatic cancer outcome. Nature.

[191] Palumbo JS (2005). Platelets and fibrin(ogen) increase metastatic potential by impeding natural killer cell-mediated elimination of tumor cells. Blood.

[192] Lo HC (2020). Resistance to natural killer cell immunosurveillance confers a selective advantage to polyclonal metastasis. Nat Cancer.

[193] Chu J (2022). Natural killer cells: a promising immunotherapy for cancer. J Transl Med.

[194] Shahzad MH (2022). Neutrophil extracellular traps in cancer therapy resistance. Cancers (Basel).

[195] Grum-Schwensen B (2015). S100A4-neutralizing antibody suppresses spontaneous tumor progression, pre-metastatic niche formation and alters T-cell polarization balance. BMC Cancer.

[196] Lambert AW (2024). Cell-intrinsic and microenvironmental determinants of metastatic colonization. Nat Cell Biol.

[197] Vandekeere A (2024). Metabolic rewiring during metastasis: the interplay between the environment and the host. Ann Rev Cancer Biol.

[198] Ramanathan RK (2019). Phase IB/II randomized study of FOLFIRINOX plus pegylated recombinant human hyaluronidase versus FOLFIRINOX alone in patients with metastatic pancreatic adenocarcinoma: SWOG S1313. J Clin Oncol.

[199] Catenacci DVT (2015). Randomized phase Ib/II study of gemcitabine plus placebo or vismodegib, a hedgehog pathway inhibitor, in patients with metastatic pancreatic cancer. J Clin Oncol.

[200] De Jesus-Acosta A (2020). Phase 2 study of vismodegib, a hedgehog inhibitor, combined with gemcitabine and nab-paclitaxel in patients with untreated metastatic pancreatic adenocarcinoma. Br J Cancer.

[201] Kindler HL (2010). Gemcitabine plus bevacizumab compared with gemcitabine plus placebo in patients with advanced pancreatic cancer: phase III trial of the Cancer and Leukemia Group B (CALGB 80303). J Clin Oncol.

[202] Elebiyo TC (2022). Reassessing vascular endothelial growth factor (VEGF) in anti-angiogenic cancer therapy. Cancer Treat Res Commun.

[203] Kindler HL (2011). Axitinib plus gemcitabine versus placebo plus gemcitabine in patients with advanced pancreatic adenocarcinoma: a double-blind randomised phase 3 study. Lancet Oncol.

[204] Zeh HJ (2020). A randomized phase II preoperative study of autophagy inhibition with high-dose hydroxychloroquine and gemcitabine/nab-paclitaxel in pancreatic cancer patients. Clin Cancer Res.

[205] Karasic TB (2019). Effect of gemcitabine and nab-paclitaxel with or without hydroxychloroquine on patients with advanced pancreatic cancer: A phase 2 randomized clinical trial. JAMA Oncol.

[206] Commisso C (2013). Macropinocytosis of protein is an amino acid supply route in Ras-transformed cells. Nature.

[207] Chung V (2025). Pembrolizumab ± paricalcitol in metastatic pancreatic cancer postmaximal cytoreduction. Oncologist.

[208] Melisi D (2019). TGFβ receptor inhibitor galunisertib is linked to inflammation- and remodeling-related proteins in patients with pancreatic cancer. Cancer Chemother Pharmacol.

[209] O’Reilly E (2022). P-22 phase III study (daNIS-2) of the anti–TGF-β monoclonal antibody NIS793 with nab-paclitaxel/gemcitabine vs nab-paclitaxel/gemcitabine alone in patients with first-line metastatic pancreatic ductal adenocarcinoma. Ann Oncol.

[210] Hu HH (2024). Roles and inhibitors of FAK in cancer: current advances and future directions. Front Pharmacol.

[211] Gardner F (2021). 1475P Results of a randomized, double-blind, placebo-controlled, phase II study of gemcitabine and nab-paclitaxel ± olaratumab in treatment-naïve patients with unresectable metastatic pancreatic cancer. Ann Oncol.

[212] Noel M et al (2020). Phase 1b study of a small molecule antagonist of human chemokine (C-C motif) receptor 2 (PF-04136309) in combination with nab-paclitaxel/gemcitabine in first-line treatment of metastatic pancreatic ductal adenocarcinoma. Invest New Drugs.

[213] Ho WJ, Jaffee EM (2021). Macrophage-targeting by CSF1/1R blockade in pancreatic cancers. Cancer Res.

[214] Hu ZI (2019). A randomized phase II trial of nab-paclitaxel and gemcitabine with tarextumab or placebo in patients with untreated metastatic pancreatic cancer. Cancer Med.

[215] Alese OB (2024). Abstract CT136: Phase Ib study of siltuximab and spartalizumab in advanced pancreatic cancer. Cancer Res.

[216] Ju Y et al (2024). Barriers and opportunities in pancreatic cancer immunotherapy. NPJ Precis Oncol.

